# Does the time of day differently impact the effects of an exercise program on postural control in older subjects? A pilot study

**DOI:** 10.1186/s13102-022-00467-5

**Published:** 2022-04-20

**Authors:** Frédéric Noé, Karim Korchi, Noëlle Bru, Thierry Paillard

**Affiliations:** 1 E2S UPPA, MEPS, Université de Pau et des Pays de l’Adour, Tarbes, France; 2 UPR APSY-v, Université de Nîmes, 5 Rue du Docteur Georges Salan CS 13019, 30021 Nîmes Cedex 1, France; 3 E2S UPPA, LMAP, CNRS, Université de Pau et des Pays de l’Adour, Anglet, France

**Keywords:** Balance, Physical activity, Aging, Chronobiology, Fall prevention

## Abstract

**Background:**

The time of day that people exercise can potentially influence the efficiency of exercises for fall prevention in older adults. The present pilot study was conducted to explore the feasibility and effects of morning versus afternoon exercising on postural control in institutionalized older adults.

**Methods:**

Nine older adults completed a 3-month multimodal exercise program in its entirety (14 participants were recruited at the beginning and were initially randomly separated into two groups). One group exercised in the morning (ME; n = 4) and the other in the afternoon (AE; n = 5). Postural control was assessed with a force platform at pre and post-intervention at the following times: 8 a.m., 11 a.m., 2 p.m. and 5 p.m.

**Results:**

Postural control significantly improved only in the AE group post-intervention. Improvements in postural control in the AE group were mainly observed in the morning.

**Conclusions:**

The afternoon would be the best period to implement exercise sessions dedicated to improve postural control in older subjects with benefits mainly observed in the morning. Further studies are needed with a larger sample in order to confirm these results.

## Introduction

Postural control is essential in daily life to maintain an upright stance without falling. It requires the central integration of sensory cues from visual, vestibular, and somatosensory receptors and the proper control of antigravity muscles [1]. Age-related deficits in either the sensory, central, or motor component of the postural control system are likely to result in an alteration of postural control which may ultimately lead to falls [2, 3]. Falls are the leading cause of accidental death among persons aged ≥ 65 years [4]. When they are not fatal, falls are associated with physical, psychological, and social consequences [5]. Regular physical activity (PA) can prevent falls or reduce their consequences by improving postural control in older adults [6–8]. The postural control system relies on sensory, central, and motor components that interact in a complex way to actively control body orientation in space and preserve balance by stabilizing the body’s centre of mass within the base of support [9]. PA programs, especially multimodal exercise programs, can positively influence each component of the postural control system, thus helping older subjects to maintain an accurate perception of body position in space and to control the movements of their centre of mass [6, 7, 10–12]. Hence, supervised PA programs are implemented for older residents in long-term care facilities as an intervention strategy to prevent falls [10, 11]. Nevertheless, due to economic and organizational constraints, the time dedicated to PA is often reduced in long-term care facilities, thus challenging the healthcare staff who tries to optimize the PA intervention modalities to maximize their effectiveness.

The time of day when people exercise is a key factor that can influence the optimization of physical performance and enhance the effectiveness of a PA program [13–18]. The effects of time of day on physical performance have been extensively studied [14, 17, 19]. While there is no clear consensus on whether there is a time-of-day effect on aerobic performance, studies about neuromuscular performance (e.g., muscle power, muscle strength, sprint) are more consistent and show that neuromuscular performance peaks in the afternoon with morning nadirs [14, 17]. The literature regarding time-of-day effects on postural control in young healthy subjects yields inconclusive results [19]. Despite a limited number of studies, evidence suggests that time-of-day effects on postural control would be more marked in older people (over 70 years old), with better postural control in the morning than in the afternoon [20–22]. Time-of-day effects on postural control in older people could relate to lower sleepiness and general fatigue in the morning than in the afternoon [22] and to cognitive function peaking in the morning in these subjects in whom cognitive processes have an increased role in postural control with advancing age [21, 23].

It is widely accepted that performance improvements are greater at the time of day at which training is conducted than at other times [13–18], thus showing that training adaptations are time-of-day dependent. Further authors also postulated that musculoskeletal adaptations would be optimized when training is performed while physical performance is peaking (i.e. at the end of the afternoon for neuromuscular performance) [13, 15, 16, 18]. The time of day when people exercise may also influence adherence and hence, the effectiveness of PA programs [24]. Knowing that postural control is better in the morning than in the afternoon in older people [20–22], the morning can be viewed as an optimal period to implement PA sessions dedicated to improve postural control in older subjects. With a reduced risk of falling due to better balance control and less fear of falling [25], older subjects are likely to feel more confident in the morning than in the afternoon, which would facilitate adherence to PA programs and would allow them to perform more challenging exercises, thus inducing more pronounced adaptations and greater improvements in postural control than when exercising in the afternoon. Nevertheless, no study has specifically investigated the effects of morning vs. afternoon training on postural control, in general and in older subjects specifically.

Hence, the aim of the present pilot study was to explore the feasibility and effects of PA performed at two different times of the day on postural control in institutionalized older adults. It was hypothesized that (1) the effectiveness of a PA program to improve postural control would be enhanced when training was performed in the morning rather than in the afternoon and (2) PA-related adaptations would be time-of-day dependent with a greater improvement of the postural control system at the time of the day that subjects have practiced than at other times.

## Materials and methods

### Participants

**Fig. 1:**
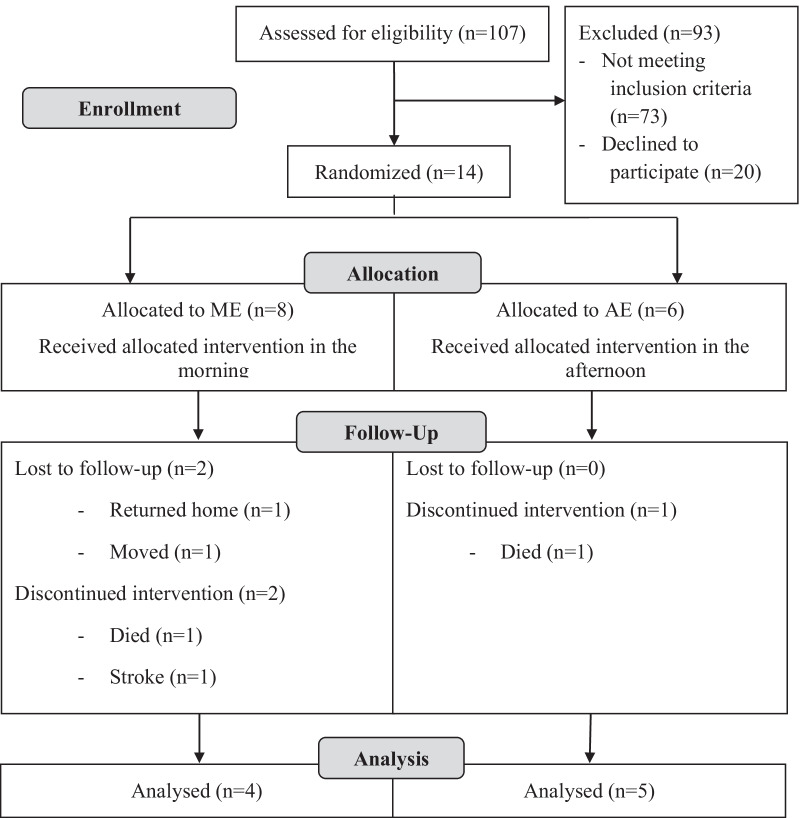
Diagram showing the flow of participants through the study

This pilot study occurred between March and June in two nursing homes located in the south of France. A flow diagram describing the recruitment of participants is presented in Fig. 1. Participants were excluded from the study protocol if a diagnosed balance control or gait disorder could impede the participants to perform balance assessments; a trauma or a severe ankylosis of the joint had occurred in the past 2 years at the hip, knee, or ankle; participants presented any lesion of the plantar surface of the foot, visual impairment, neurological, mental or cognitive disorders, pulmonary or cardiac problems (e.g. coronary artery disease, myocardial infarction, congestive heart failure, permanent or paroxysmal heart rhythm disturbances, poorly controlled hypertension). All of these criteria are known to restrict or preclude the participation in physical exercise [26]. Verbal and written consent was obtained from the residents or from their legal guardian or family member before starting the experiment.

Of the randomized 14 participants at baseline, 9 participants—5 women and 4 men aged 79 to 102 years [medians (IQR): age 87.5 (12.5) years old, height: 166.0 (10.3) cm, weight: 66.3 (9.9) kg] completed the study and were included in the analysis (Fig. 1). They were randomly separated into two groups: the ME (n = 4) and the AE (n = 5) groups (see Table 1 for the characteristics of the two groups), whose members were involved in a multimodal exercise program that was performed exclusively in the morning (ME group) or exclusively in the afternoon (AE group). The study was conducted in accordance with the Declaration of Helsinki, and the protocol was approved by the Ethics Committee of the Université de Pau et des Pays de l’Adour.

**Table 1:** Characteristics of the subjects from ME and AE groups at baseline [medians (IQR)]

Variables	Morning exercise (n = 4)	Afternoon exercise (n = 5)
Age (years)	81.0 (5.8)	89 (7.3)
Height (cm)	164 (9.5)	165 (8.8)
Weight (kg)	64.4 (13.2)	67.9 (7.5)

No significant differences were noted for any of these variables between ME and AE groups

### Exercise intervention

The multimodal exercise program consisted of 60 min of supervised exercise sessions performed three times a week for 3 months. It included walking, strength, flexibility, coordination, and balance training. Each session began with a 10-min warm-up routine. Then participants had to perform a 20-min walk on an inside walking trail, during which they had to avoid obstacles by changing direction. They were also encouraged to walk fast to reach moderate breathlessness but not exhaustion. Afterwards, strength, balance, and coordination training exercises were interspersed during a 20-min period. Strength training was conducted with sit-to-stand exercises while using chairs at different heights (55 cm, 51 cm, 47 cm, and 43 cm) to modulate intensity. Participants also had to perform single-leg extensions with weights on their ankles and calf extensions from a sitting position with weights on their thighs. Balance training consisted of keeping balance for 20–30 s or throwing a ball into a target with different positions of the feet. Coordination exercises took the form of ball games as well as imitation exercises. Finally, a 10-min stretching routine was held at the end of the session as a cool-down phase when attention was focused on breathing. A demonstration of each exercise was carried out by using adjusted communication with simple language. Exercises were individualized according to the constraints and attributes of each participant and began at very light intensities. As the exercise intervention progressed, participants were subjected to motor “overload” achieved by a slow and gradual increase of exercise intensity and complexity [6]. The ME group exercised between 10 a.m. and 12 p.m. and the AE group exercised between 2 p.m. and 4 p.m. Both the ME and AE groups conducted similar training sessions. Each exercise session was supervised by the same person.

### Measurements

Postural control was assessed by a “blinded-to-treatment” allocation investigator in the early morning at 8 a.m., in the late morning at 11 a.m., in the early afternoon at 2 p.m. and in the late afternoon at 5 p.m. to compare postural control before (PRE) and after (POST) the 3-month exercise intervention. Participants performed these four testing sessions in a random order during the same week. They were instructed to sway as little as possible for 30 s when standing on stable ground from a bipodal stance with their eyes closed. Standing with their eyes-closed is a challenging task, which might better detect time-of-day related changes in postural control [19] and improvements in postural control due to exercise interventions in older adults [27]. Participants stood barefoot in the middle of a force platform (Stabilotest^©^, Techno Concept™, Mane, France) with their arms along the body and their feet at an angle of 30° with a 2 cm spacing between the heels. Data acquisition was always preceded by a familiarization trial. The centre of pressure displacements (COP) were recorded at a 40 Hz sampling frequency. COP surface area (area: 90% confidence ellipse), mean COP velocity along the medio-lateral (mean velocity ML) and anterior–posterior (mean velocity AP) axes were calculated [28].

### Statistical analysis

After testing the normality of the dataset with the Shapiro–Wilk test, nonparametric tests were applied as most of the variables did not meet the normality assumption. PRE and POST measurements were compared within each group at different assessment times (8 a.m., 11 a.m., 2 p.m., 5 p.m.) with Wilcoxon signed-rank tests in order to test the effect of the PA program and to characterize at which time-of-day this effect occurred. The relative difference of each centre of pressure parameter between PRE and POST measurements [ΔPre-Post (%) = 100 * (POST − PRE) ÷ PRE] was also calculated. Relative difference is an easily interpretable descriptor to characterize PRE-POST evolutions and between-group changes induced by the PA program while limiting the influence of intra-group heterogeneity. ΔPre-Post was compared between the ME and AE groups with a Wilcoxon–Mann–Whitney test to test for differences in PRE-POST evolution between each group. The significance level was set at *P* < 0.05. Statistical analyses were performed with R statistical software [29].

## Results

Table 2 presents the median and interquartile range of postural control variables in the ME and AE groups in PRE and POST conditions. In the AE group, two COP parameters reached significance between PRE and POST values: area and mean velocity ML significantly decreased from PRE to POST at 8 a.m. (V = 21, *P* = 0.03; V = 21, *P* = 0.03 respectively). In the ME group, no significant differences were observed between PRE and POST conditions. Significant differences between the groups were detected for ΔPre-Post at 8 a.m. (COP area; W = 2, *P* = 0.04) and at 2 p.m. (COP mean velocity ML; W = 0, *P* = 0.01): the AE group was characterised by negative values ΔPre-Post (i.e. participants benefited from the PA program to improve postural control) whereas the ME group was characterized by positive values ΔPre-Post (i.e. participants did not benefit from the PA program to improve postural control).

**Table 2:** PRE and POST values of centre of pressure parameters [median (IQR)] at each instant (8 a.m., 11 a.m., 2 p.m. and 5 p.m.) in both groups (ME and AE)

Assessment time	COP parameters	Group	PRE	POST	ΔPre-Post
8 a.m.	Area (mm^2^)	ME	549.3 (572.5)	520.5 (357.0)	+ 43.6 (43.2) %
		AE	544.3 (182.7)	336.2 (208.4)*	− 40.5 (35.2) %^**†**^
	Mean velocity ML (mm s^−1^)	ME	13.0 (10.9)	11.8 (8.1)	+ 21.4 (24.1) %
		AE	13.3 (3.1)	13.2 (2.5)*	− 3.6 (5.5) %
	Mean velocity AP (mm s^−1^)	ME	12.9 (16.7)	17.9 (12.9)	+ 32.3 (30.6) %
		AE	15.5 (8.7)	20.6 (15.6)	+ 20.9 (32.8) %
11 a.m.	Area (mm^2^)	ME	861.0 (1411.6)	465.3 (592.5)	− 49.7 (37.7) %
		AE	484.5 (248.0)	449.2 (360.7)	− 19.2 (26.3) %
	Mean velocity ML (mm s^−1^)	ME	12.8 (16.2)	10.0 (4.7)	− 20.2 (33.6) %
		AE	12.6 (5.1)	11.0 (3.4)	− 4.6 (49.8) %
	Mean velocity AP (mm s^−1^)	ME	12.9 (21.7)	17.3 (12.9)	+ 17.9 (74.4) %
		AE	18.4 (5.7)	18.3 (10.0)	+ 5.8 (15.3) %
2 p.m.	Area (mm^2^)	ME	675.4 (1136.1)	632.1 (1215.4)	+ 50.5 (54.4) %
		AE	558.66 (329.4)	344.9 (90.6)	− 22.3 (52.3) %
	Mean velocity ML (mm s^−1^)	ME	11.2 (9.2)	13.8 (13.9)	+ 39.7 (27.2) %
		AE	13.0 (3.1)	11.4 (2.5)	− 5.7 (19.1) %^**†**^
	Mean velocity AP (mm s^−1^)	ME	15.7 (18.0)	17.1 (15.1)	+ 12.2 (27.5) %
		AE	19.4 (12.6)	19.9 (15.2)	− 3.9 (4.4) %
5 p.m.	Area (mm^2^)	ME	762.0 (689.3)	524.8 (380.3)	− 31.9 (7.2) %
		AE	514.8 (235.2)	575.2 (644.6)	− 0.8 (80.2) %
	Mean velocity ML (mm s^−1^)	ME	14.4 (7.0)	13.1 (9.1)	− 3.3 (11.8) %
		AE	12.3 (4.0)	13.9 (4.6)	− 0.1 (36.8) %
	Mean velocity AP (mm s^−1^)	ME	16.1 (16.2)	18.8 (13.1)	+ 16.0 (17.6) %
		AE	22.9 (13.3)	23.9 (14.1)	+ 12.1 (53.7) %

ME = morning exercise group; AE = afternoon exercise group; PRE = pre-exercise intervention values; POST = post-exercise intervention values. ΔPre-Post: relative difference between PRE and POST measurements [ΔPre-Post = 100 * (POST − PRE) ÷ PRE]; negative and positive values characterize participants who benefit from the PA program—or not—to improve postural control

*Illustrates a significant difference between PRE and POST values

^**†**^Illustrates a significant between-groups difference (*P* < 0.05)

## Discussion

This pilot study investigated the feasibility and effects of a 3-month PA program performed either in the morning or in the afternoon on postural control in institutionalized older adults. The PA program was well received, likely due to the individualization and the gradual progression of the training, which provided adapted challenges and facilitated social interaction among residents who took part in the program. Indeed, older people participated in all sessions and no-one dropped out during the program but for major health issues (death or stroke). It should be noted that participants from the AE group were not disturbed by the afternoon exercise sessions, which were scheduled after a significant rest period (at least 1 h) after lunch.

Despite our initial hypothesis that the effectiveness of a PA program to improve postural control can be enhanced when training was performed in the morning rather than in the afternoon, our results showed that postural control significantly improved only when training was performed in the afternoon. While investigating the time-of-day effects of an acute resistance exercise session on the hypertrophic response, Burley et al. [13] showed that training in the late afternoon provided a superior anabolic environment to optimize the muscle hypertrophic response, thanks to a diminished catabolic process (reduced cortisol concentrations) and increased anabolic signalling (elevated insulin-like growth factor-binding protein-3 levels). By contrast, exercising in the morning with high cortisol levels resulted in a catabolic environment attenuating protein synthesis [13]. Despite non-univocal results about the long-term beneficial impact of afternoon versus morning training [30], these more favourable acute effects of afternoon training due to diurnal variation in hormonal levels could explain the larger chronic musculoskeletal adaptations previously reported with afternoon training [16, 17]. Similarly, the beneficial effect of afternoon training on postural control reported in the present study (characterized by the significant decrease of further COP parameters) could be explained by the optimized musculoskeletal adaptations induced by afternoon PA, thus leading to a greater impact on the motor component of the postural control system. Optimizing the neuromuscular impact of PA programs by generating muscular hypertrophy is critical for older adults to counteract the age-related decline in muscle mass and muscle function which strongly affects postural control [31, 32]. Our results therefore suggest that training in the afternoon (and not necessarily when physical performance is peaking) would induce superior adaptations to morning training, thus confirming the idea of optimized musculoskeletal adaptations with afternoon training [13, 15, 16, 18].

Physical performance usually improves in young subjects at the time of training [14]. Our results surprisingly illustrate that exercising in the afternoon rather produced improvement of older adults’ postural control in the morning since PRE and POST comparisons within each group were only significant in the AE group at 8 a.m. The time of day has a substantial influence on postural control in the elderly, with postural control being better in the morning than in the afternoon in these subjects [20–22]. Explanations for the better postural control in the morning than in the afternoon in older subjects could relate to lower sleepiness and general fatigue in the morning than in the afternoon [22]. Moreover, postural control in older people relies more on cognitive function with a greater cortical involvement than young subjects [33]. Knowing that cognitive function is peaking early in human circadian rhythms and is worsening through the day in older individuals [21, 23, 34], the better postural control in the morning than in the afternoon in older people could also be related to the daily fluctuation of cognitive function. Hence, the morning can be considered as the most favourable time of day to perform postural tasks for older people. The improvement of older adults’ postural control in the morning following the 3-month PA program performed in the afternoon could therefore characterise a singular positive training transfer effect [35]. Afternoon training might have enhanced the motor component of the postural control system by optimising musculoskeletal adaptations. Nevertheless, the positive training transfer effect (due to afternoon training) on the entire postural control system would have rather occurred at the most favourable time of day for postural control, i.e., in the morning.

Limitations of the current research are acknowledge. The first and main limitation of this pilot study was the small sample size. Therefore, caution in the generalization of these results has to be observed and future studies have to be conducted with a larger sample size to confirm these results. Secondly, although very old participants took part in this study, there were large differences in age between them that could have influenced the results. Hence, future studies should also address the issue of inter-individual differences in responses to exercise programs performed at different times of the day, by specifying in particular the potential influence of age and gender differences.

## Conclusions

From a practical point of view, results from the present pilot study suggest that the afternoon would be the best period to implement exercise sessions dedicated to improve postural control in older adults. Nevertheless, with a higher risk of falling in the afternoon due to a worsened postural control than in the morning [20–22], training in the afternoon with older adults should be conducted with great caution. Hence, supervised PA training programs conducted by health clinicians or PA professionals would be preferred to unsupervised programs.

## Data Availability

The datasets used and/or analysed during the current study are available from the corresponding author on reasonable request.
